# Effect of a brief physical activity-based presentation by a former patient for men treated with radical prostatectomy for prostate cancer: a mixed methods pilot study

**DOI:** 10.1007/s00520-020-05455-4

**Published:** 2020-04-22

**Authors:** Louis Fox, Theresa Wiseman, Declan Cahill, Louisa Fleure, Janette Kinsella, Emily Curtis, Nicola Peat, Mieke Van Hemelrijck

**Affiliations:** 1 Translational Oncology and Urology Research, King’s College London, London, UK; 2 Guy’s Hospital, 3rd floor, Bermondsey Wing, Great Maze Pond, London, SE1 9RT UK; 3 Applied Health Research, The Royal Marsden NHS Foundation Trust, London, UK; 4 Urology Surgery, The Royal Marsden NHS Foundation Trust, London, UK; 5 Urology Services, Guy’s and St Thomas’ NHS Foundation Trust, London, UK; 6 Physiotherapy, Guy’s and St Thomas’ NHS Foundation Trust, London, UK

**Keywords:** Physical activity, Prostate, Behaviour, Exercise, Oncology, Rehabilitation, Cancer, Prostatectomy, Self-management, Quality of life

## Abstract

**Purpose:**

Existing research indicates that physical activity (PA) is beneficial to men with prostate cancer (PCa). We examined the potential of a single-contact peer-support-based behavioural intervention to promote PA engagement in men treated for PCa.

**Methods:**

A mixed methods design was employed, comprising a two-arm pragmatic trial and semi-structured interviews. The intervention was a 10-min PA-based presentation by a former patient, delivered in group seminars that are provided for patients as standard care. Seminars were alternately allocated to (a) cancer exercise specialist talk + patient speaker talk or (b) cancer exercise specialist talk only. Self-reported PA, exercise motivation, quality of life, fatigue and clinical and demographic characteristics were obtained from *n* = 148 (intervention: *n* = 69; control: *n* = 79) patients immediately prior to the seminar, and at follow-up ≈ 100 days later. Data were analysed using ANCOVA models and *χ*^2^ tests. Fourteen semi-structured interviews with intervention participants, which explored how the intervention was experienced, were analysed using a grounded theory-style approach.

**Results:**

The intervention had no significant effect on quantitatively self-reported PA (*p* = 0.4). However, the intervention was statistically and clinically beneficial for fatigue (*p* = 0.04) and quality of life (*p* = 0.01). Qualitative analysis showed that the intervention was beneficial to psychological wellbeing and some participants had increased intention to engage in PA as a result of the intervention.

**Conclusions:**

A brief one-off PA-based presentation for men with PCa, delivered by a former patient alongside cancer exercise specialist advice, may result in clinically significant benefits to quality of life and may influence PA intention in certain individuals.

**Electronic supplementary material:**

The online version of this article (10.1007/s00520-020-05455-4) contains supplementary material, which is available to authorized users.

## Introduction

Prostate cancer (PCa) is the second most common cancer in men, with 47,000 cases diagnosed every year in the UK [1]. It is well established that engagement with physical activity (PA) is beneficial to men diagnosed with PCa [2]. Suggested benefits of PA include improvements to cancer-specific quality of life [3], improvements to cancer-specific fatigue [4], and amelioration of certain treatment side effects—particularly physiological ones commonly accompanying hormonal therapy [5]. There exists epidemiological evidence linking postdiagnosis PA with slowed disease progression [6] and cancer-specific survival [7–11].

Despite the known benefits and NICE guidelines recommending offering 12 weeks of supervised exercise to patients [12], many men with PCa are not meeting the advised levels of regular PA for people with cancer [13]. The factors determining men’s likelihood of engaging with PA following a diagnosis are complex, including financial governance; organizational culture, organizational processes, availability of social support, the availability of relevant expertise, effects of diagnosis and treatment, patient capability, and patient motivation [14, 15]. Here, we focus primarily on patient motivation, reporting a mixed methods pilot study which examined the potential of a brief peer-support-based behavioural intervention, provided alongside standard care, to promote PA engagement in men treated for PCa.

This pilot study has been undertaken in the context of existing National Health Service (NHS) care pathways for men with PCa. The care pathways utilise educational seminars for patients, the purpose of which is to disseminate information to patients undergoing treatment for PCa, in a manner which is comprehensive but also economically efficient [16]. This patient seminar format was the mode of delivery for the intervention.

The primary aim of this exploratory pilot study was to examine whether a brief peer support-based intervention, in which patients are persuaded of the merits of PA by a former patient, can promote PA behaviour in men who have received radical prostatectomy for localised prostate cancer. The secondary aim was to examine whether such an intervention can be beneficial to these patients’ quality of life.

## Methods

The protocol for the quantitative aspects of this study has been published previously [17]. A full summary of all methods is provided in Appendix 1 (see online supplement). A summary of the key aspects of the methods is provided here.

### Study design

This was a mixed methods study, comprising a pragmatic trial [18] and qualitative interviews, in a fully mixed, sequential (quantitative → qualitative), equal status design, according to Leech and Onwuegbuzie’s typology of mixed methods studies [19]. The mode of delivery for the intervention was educational seminars, covering various topics including diet, fluid intake, psychological advice, and PA, which are delivered in a urology clinic as part of standard NHS care for men undergoing radical prostatectomy. The pragmatic trial design alternately allocated educational seminars to either a control condition, consisting of the existing seminar format, or an intervention condition, existing of a new seminar format containing the intervention. Patients attending the standard or intervention seminars were approached with optional questionnaires to complete, and the final sample was opportunistic, comprising all patients who opted to return data at both study time points.

### Intervention

Data were obtained from two study sites, at which the intervention was applied in differing contexts. At site A, the intervention was delivered via a posttreatment seminar, which all men undergoing radical prostatectomy were invited to, but at which attendance was optional. At site B, the intervention was delivered in a pretreatment seminar, which is generally mandatory before undergoing radical prostatectomy. At each site, individual seminars were allocated into one of the two formats^1^:

1. Control seminar. Patients heard a 20-min PA presentation by a cancer exercise specialist. This presentation covered the evidence that PA is beneficial to people with cancer, official PA recommendations, what constitutes moderate and vigorous PA, assurance that PA is safe for them and advice on how to integrate PA into everyday life.
2. Intervention seminar. Patients heard the above presentation, immediately followed by a 10-min PA presentation by a non-clinician previously treated for locally advanced PCa. The presentation was delivered in a narrative style, focusing on his personal story, describing his positive experience of PA, within the context of his medical history of treatment for PCa with radical prostatectomy and hormone therapy.

### Participants

Participants were 148 men (*n* = 79 control; *n* = 69 intervention) who had received radical prostatectomy (i.e., curative therapy) for localised prostate cancer (see Table 1). Fifteen men who experienced the intervention were approached for qualitative interview, of which fourteen were interviewed.

**Table 1:** Clinical and demographic characteristics of study sample

Mean (SD)			Control	Intervention	Total	*p*
			(*n* = 79)	(*n* = 69)	(*n* = 148)	
	Age (years)		63.8 (6.8)	63.4 (6.4)	63.6 (6.6)	0.69
	Days elapsed between baseline and T_1_		101.8 (17.4)	98.4 (17.7)	100.2 (17.6)	0.24
*n* (%)						p
	Study site	Site A	36 (45.6)	35 (50.8)	71 (48)	0.53
		Site B	43 (54.4)	34 (49.2)	77 (52)	
Clinical characteristics	Had lymph node dissection?	Yes	6 (7.6)	4 (5.8)	10 (6.8)	0.75
		No	73 (92.4)	65 (94.2)	138 (93.2)	
	Had bladder neck reconstruction?	Yes	17 (21.5)	20 (29)	37 (25)	0.34
		No	62 (78.5)	49 (71)	111 (75)	
	On hormone therapy?	Yes	2 (2.5)	1 (1.4)	3 (2)	0.64
		No	77 (97.5)	68 (98.6)	145 (98)	
	Hypertension?	Yes	21 (26.6)	18 (26.1)	39 (26.4)	0.95
		No	58 (73.4)	51 (73.9)	109 (73.6)	
	Other non-hypertension comorbidity?	Yes	9 (11.4)	9 (13)	18 (12.2)	0.76
		No	70 (88.6)	60 (87)	130 (87.8)	
Demographic characteristics	Employment status	Working full time	34 (43)	34 (49.3)	68 (45.9)	0.18
		Working part time	12 (15.2)	4 (5.8)	16 (10.8)	
		Not working	33 (41.8)	31 (44.9)	64 (43.2)	
	Marital status	Partnered	73 (92.4)	63 (91.3)	136 (91.9)	0.80
		Not partnered	6 (7.6)	6 (8.7)	12 (8.1)	
	Education status^a^	University/college degree	40 (50.6)	33 (48.5)	73 (49.7)	0.80
		No degree	39 (49.4)	35 (51.5)	74 (50.3)	
	Ethnicity	White	66 (83.5)	56 (81.2)	122 (82.4)	0.43
		Black	7 (8.9)	10 (14.5)	17 (11.5)	
		Asian	6 (7.6)	3 (4.3)	9 (6.1)	

^a^*n* = 1 missing data

The inclusion criteria were:

- Diagnosis of localised prostate cancer.
- Received radical prostatectomy for prostate cancer at one of the two sites studied.
- Attended a posttreatment (site A) or pretreatment (site B) educational seminar.
- [Qualitative interviews] Attended an intervention seminar.

Patients were excluded if:

- They were unable to speak English.
- They had a visual or hearing impairment.
- They experienced postoperative complications that resulted in a deviation from standard postoperative follow-up procedures.

### Primary outcome: self-reported PA

The primary outcome was self-reported PA. This was measured at baseline (immediately prior to the seminar) and at T_1_, which was either 90 days after the seminar (site A); or 90 days after undergoing radical prostatectomy (site B). Self-reported PA was measured using the Short Questionnaire to Assess Health-Enhancing Physical Activity (SQUASH) [21]. The SQUASH is a brief questionnaire that asks respondents to report how much PA they have done in the past week. For analysis, responses were converted to metabolic task equivalent (MET) minutes.

### Secondary outcomes

#### Quality of life

Quality of life was measured at both baseline and T_1_ using the EQ-5D-5L [22]. The EQ-5D-5L contains two parts. The first part asks respondents to report the extent to which they are experiencing problems on five dimensions: *mobility*, *self-care*, *usual activities*, *pain/discomfort* and *anxiety/depression*. The second part, the visual analogue scale (VAS), asks participants to report how healthy they feel, from 0 (worst health you could imagine) to 100 (best health you could imagine). This produces six scores: one for each dimension, and one for the VAS.

#### Fatigue

Fatigue was measured at both baseline and T_1_ using the FACIT-Fatigue scale [23]. This scale contains 13 statements about fatigue and asks respondents to report how true each statement is for them currently, on a 5-point scale; the maximum fatigue score is 52.

#### Exercise motivation

Exercise motivation was measured at both baseline and T_1_ using the Behavioural Regulations in Exercise Questionnaire-3 (BREQ-3) [24]. This questionnaire contains 24 items and examines six aspects of motivation: *amotivation*, *external regulation*, *introjected regulation*, *identified regulation*, *integrated regulation* and *intrinsic motivation*.

### Qualitative methods summary

#### Interview participants

Fifteen men who had attended an intervention seminar, and were happy to be contacted regarding an interview, were successfully contacted. Fourteen of these were interviewed (one did not attend his appointment). The ethnicities of the interviewees were broadly representative of the wider sample (see Table 1). Some were retirees; others were still working.

#### Interview procedures

Interviews were semi-structured and lasted approximately 40 min. They took place in a research office based on the hospital site (participants were offered flexibility on the interview location, but all opted to visit the hospital). The interviews followed a topic guide, which had been developed by two authors (LF and TW) and aimed to gain insight into PA history and how the intervention was experienced. Quantitative questionnaire responses were used to prompt discussion. Interviews were audio recorded and transcribed.

## Results

### Quantitative results

#### Response rate

**Fig. 1:**
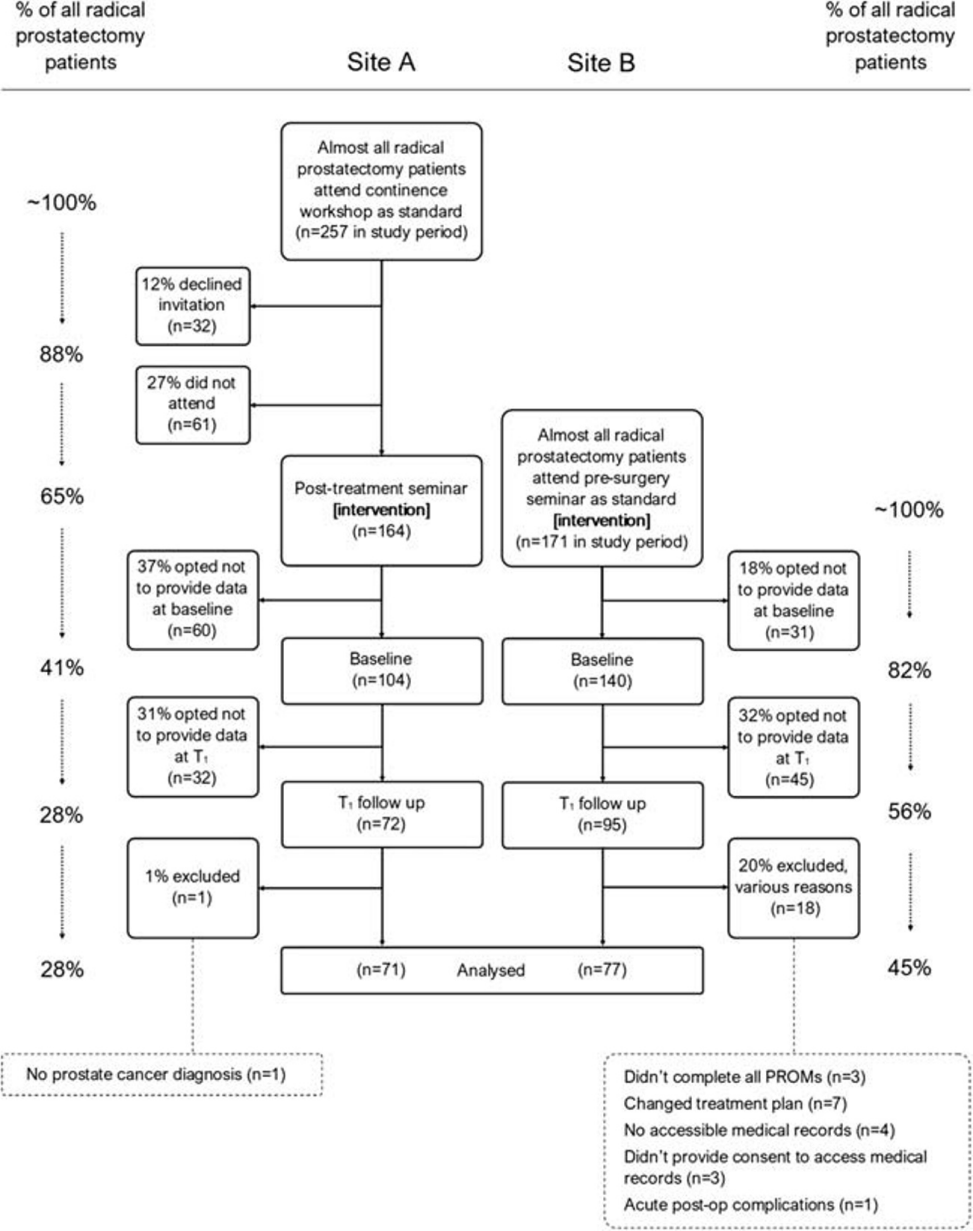
Flow diagram showing the proportion of eligible patients that provided data for the analysis

Figure 1 shows the proportion of eligible patients that opted to provide data at each time point. Across the two study sites, approximately one third of patients who received radical prostatectomy in the study time period provided data that was included in the analysis.

#### Baseline characteristics

Table 1 shows clinical and demographic characteristics of the final analysis sample (*n* = 148). Experimental groups did not differ on any clinical or demographic characteristics. The groups also did not differ at baseline for any of the patient-reported outcome measures, except for the ‘pain/discomfort’ dimension of the EQ-5D-5L, on which intervention group participants were overrepresented amongst those reporting ‘slight problems’ (as opposed to ‘no problems’) (see Table 2).

**Table 2:** Baseline comparison of experimental groups on patient-reported outcome measures

		Mean (SD)	Control	Intervention	Total	*p*
			(*n* = 79)	(*n* = 69)	(*n* = 148)	
BREQ-3		Amotivation	0.22 (0.56)	0.26 (0.55)	0.24 (0.55)	0.66
		External regulation	0.65 (0.79)	0.72 (0.93)	0.68 (0.85)	0.65
		Introjected regulation	1.88 (1.09)	1.85 (1.11)	1.87 (1.1)	0.84
		Identified regulation	3.18 (0.86)	3.13 (0.75)	3.16 (0.81)	0.73
		Integrated regulation	2.45 (1.36)	2.65 (1.25)	2.54 (1.31)	0.36
		Intrinsic motivation	2.81 (1.04)	2.83 (1.09)	2.82 (1.06)	0.92
		FACIT-Fatigue	6.3 (7.3)	6.1 (6.8)	6.2 (7)	0.83
		EQ-5D-5L Visual Analogue Scale (QoL)	83 (11.4)	80.5 (14.1)	81.8 (12.7)	0.23
		Incontinence pads used per day (T_1_)	1 (1.4)	1.1 (1.2)	1.1 (1.3)	0.54
		Self-reported MET minutes	3207 (2949)	3251 (3274)	3228 (3095)	0.93
		*n* (%)				
EQ-5D-5L quality of life	*(no problems)*	Level 1	70 (88.6)	59 (85.5)	129 (87.2)	0.58
		Level 2	7 (8.9)	8 (11.6)	15 (10.1)	
	Mobility	Level 3	1 (1.3)	2 (2.9)	3 (2)	
		Level 4	1 (1.3)	0	1 (0.7)	
	*(extreme problems)*	Level 5	0	0	0	
	Self-care	Level 1	79 (100)	69 (100)	148 (100)	–
		Level 2	0	0	0	
		Level 3	0	0	0	
		Level 4	0	0	0	
		Level 5	0	0	0	
	Usual activities	Level 1	68 (86.1)	59 (85.5)	127 (85.8)	0.78
		Level 2	9 (11.4)	9 (13)	18 (12.2)	
		Level 3	2 (2.5)	1 (1.4)	3 (2)	
		Level 4	0	0	0	
		Level 5	0	0	0	
	Pain/discomfort	Level 1	57 (72.2)	40 (58.0)	97 (65.5)	0.05
		Level 2	17 (21.5)	25 (36.2)	42 (28.4)	
		Level 3	3 (3.8)	4 (5.8)	7 (4.7)	
		Level 4	2 (2.5)	0	2 (1.4)	
		Level 5	0	0	0	
	Anxiety/depression	Level 1	55 (69.6)	42 (60.9)	97 (65.5)	0.40
		Level 2	19 (24.1)	20 (29)	39 (26.4)	
		Level 3	5 (6.3)	7 (10.1)	12 (8.1)	
		Level 4	0	0	0	
		Level 5	0	0	0	

*MET* metabolic task equivalent, *QoL* quality of life

#### Primary outcome: self-reported PA

Results for continuous outcomes are shown in Table 3. There was no significant difference in changes in self-reported PA between the groups.

**Table 3:** Unadjusted means and *p* values from ANCOVA tests of continuous outcome variables (*n* = 148)

	Baseline		T_1_		Change T_0_-T_1_		Interaction
	*M* (SD)		*M* (SD)		*M* (SD)		(group × time)
	Control	Intervention	Control	Intervention	Control	Intervention	*p*
Self-reported MET minutes^a^	3207 (2949)	3251 (3274)	3915 (4120)	3505 (4120)	+ 708 (3813)	+ 254 (3127)	0.38
BREQ-3: Amotivation^a^	0.22 (0.56)	0.26 (0.55)	0.16 (0.47)	0.2 (0.44)	− 0.06 (0.57)	− 0.06 (0.5)	0.88
BREQ-3: External regulation^a^	0.65 (0.79)	0.72 (0.93)	0.51 (0.82)	0.58 (0.87)	− 0.14 (0.69)	− 0.14 (0.69)	0.95
BREQ-3: Introjected regulation^a^	1.88 (1.09)	1.85 (1.11)	1.68 (1.17)	1.67 (1.18)	− 0.20 (0.89)	− 0.18 (1.21)	0.86
BREQ-3: Identified regulation^a^	3.18 (0.86)	3.13 (0.75)	3.07 (0.87)	3.0 (0.84)	− 0.11 (0.66)	− 0.13 (0.61)	0.81
BREQ-3: Integrated regulation^a^	2.45 (1.36)	2.65 (1.25)	2.41 (1.37)	2.38 (1.33)	− 0.04 (0.75)	− 0.27 (0.99)	0.12
BREQ-3: Intrinsic motivation^a^	2.81 (1.04)	2.83 (1.09)	2.63 (1.08)	2.65 (1.14)	− 0.18 (0.74)	− 0.18 (0.87)	0.92
ED-5D-5 L Visual Analogue Scale^a^	83 (11.4)	80.5 (14.1)	81.7 (17.5)	84.5 (11.4)	− 1.3 (16.4)	+ 4 (12.4)	0.04
FACIT-fatigue^b^	6.3 (7.3)	6.1 (6.8)	9.7 (8.8)	6.7 (7.0)	+ 3.4 (6.6)	+ 0.6 (5.2)	0.01

*df* (1, 145)

*MET* metabolic task equivalent, *PA* physical activity

^a^Covariates: study site

^b^Covariates: study site; time of year

#### Secondary outcomes: continuous variables

Fatigue and subjective quality of life scores both favoured the intervention (Table 3). Post hoc analyses showed that the effect on the EQ-5D-5L VAS was driven predominantly by men at site B (who underwent radical prostatectomy between time points), and that intervention participants were less likely to cross the clinically significant threshold for deterioration on both fatigue (*χ*^2^ = 3.5, *p* = 0.06) and VAS score (χ^2^ = 4.2, *p* = 0.04). There was no significant difference in changes in exercise motivation between the groups.

#### Secondary outcomes: categorical variables

Results for the five dimensions of the EQ-5D-5L are shown in Table 4. *χ*^2^ tests showed that men exposed to the intervention were significantly more likely to improve, and less likely to deteriorate, between baseline and T_1_ on the dimensions of *usual activities*, *pain/discomfort* and *anxiety/depression*. Additional *χ*^2^ tests showed that whether individuals improved, or deteriorated, was not associated with *Study site* on any of the five dimensions.

**Table 4:** Frequency distributions of changes in quality of life dimensions between baseline and T_1_, measured using the EQ-5D-5L, with percentages and *χ*^2^ tests (*n* = 148)

	Improved	Stayed the same	Deteriorated	*χ*^2^	*p*
EQ-5D-5L dimensions					
Mobility					
Control	4 (5.1)	69 (87.3)	6 (7.6)	3.46	0.18
Intervention	8 (11.6)	59 (85.5)	2 (2.9)		
Self-care					
Control	0	79 (100)	0	2.32	0.13
Intervention	0	67 (97.1)	2 (2.9)		
Usual activities					
Control	3 (3.8)	56 (70.9)	20 (25.3)	7.93	0.02
Intervention	8 (11.6)	54 (78.3)	7 (10.1)		
Pain/discomfort					
Control	10 (12.7)	54 (68.4)	15 (19.0)	7.95	0.02
Intervention	21 (30.4)	41 (59.4)	7 (10.1)		
Anxiety/depression					
Control	6 (7.6)	59 (74.7)	14 (17.7)	6.68	0.04
Intervention	14 (20.3)	49 (71.0)	6 (8.7)		

Further *χ*^2^ tests were performed, on the dimensions of ‘pain/discomfort’ and ‘anxiety/depression’, to account for discrepancy between groups at baseline on these dimensions (see Table 2). These tests included only men who had indicated ‘slight problems’ at baseline, hence ensuring that each group had equal opportunity to improve. The observed relationships were maintained (*pain/discomfort*: *χ*^2^ = 9.5, *p* < 0.01; *anxiety/depression*: *χ*^2^ = 8.1, *p* < 0.02).

#### Qualitative results

Emergent themes from the interviews were (1) reassurance about the future from someone with experience, (2) a sense of social solidarity, (3) positive disposition amongst the impressionable and (4) social comparison and attitude renewal. The latter two themes relate to exercise behaviour.

##### Reassurance about the future from someone with experience

Some participants spoke of a sense of dread that had descended on them upon being diagnosed with PCa.

“I’m one of those people who … I can’t see myself dying. [The diagnosis] changes everything, you start planning your own funeral, it’s such a shock.” (Participant 3)

“While you’re in it … you don’t think you’re ever going to get through it.” (Participant 7)

However, many participants noted that the intervention helped to alleviate this somewhat. This was sometimes framed by participants as attaining some degree of vision of hope for the future.

“He persuaded me that there is hope, there is a future, you can make it.” (Participant 1)

This framing is illustrated well by metaphors used by some of the participants:

“I knew there was light at the end of the tunnel because I could see it increase [during the talk].” (Participant 1)

“Yes, it did affect me, in the sense that it made me… How can I say? It gave me a light at the end of the tunnel.” (Participant 2)

This seemed to be linked to the fact that the speaker was a credible source, as someone who had been through the experience…

“It was quite nice when they have someone rather than just a nurse, a clinician or someone like that, to have a real-life story. Then you come away thinking, ‘I’m going to get through this.’” (Participant 7)

“He’d gone through it himself … You can see what life is after and that.” (Participant 9)

…and was an experience characterised by positive affect:

“Fundamentally he was emphasising on the importance of being positive about your treatment … it was his sense of positivity that I got out of that, which was nice.” (Participant 4)

“You hear someone speak like that, and it was a genuine talk, then that’s it, you know, you really remove, mentally– any negatives you were thinking, it removed it from your mind.” (Participant 6)

##### A sense of social solidarity

Many participants suggested that a sense of relatability and solidarity between patients who have been through the same experience was a valuable resource to integrate into the patient pathway, and that clinical staff are not necessarily able to provide this type of support.

“Forgive me, I mean, most of [the cancer nurses] are, as you said earlier, are female. They don’t understand. Well, they do understand, but they’ve not been through it.” (Participant 12)

“Your voluntary workers who work in the clinics … that’s what got me through. I think if it wasn’t for these guys, I might’ve been on edge thinking, like, ‘What have I done?’” (Participant 10)

It was important to some participants to be provided with a sense that they were not alone, or to have feelings of anxiety alleviated.

“The seminars give you the confidence and assure you, you are not alone.” (Participant 1)

“I don’t know… what is it? Reassuring I suppose that other people have been through the same thing and, well, they’re still alive … [Interviewer: Do you think it alleviates anxiety?] Yes, yes.” (Participant 11)

One participant even said that he did not necessarily believe clinical advice from his consultant, but that he did believe advice from the patient speaker:

“You don’t necessarily believe what the doctors and clinicians are saying … Then when you see the chap who’s giving you the real-life story you think, ‘Actually, this is something that’s coming from his mouth that he’s experienced.’” (Participant 7)

##### Positive disposition amongst the impressionable

Some of the interviewees drew a direct causal link between being exposed to the intervention and subsequently increasing their PA behaviour:

“I made my decision from that moment to improve.” (Participant 1)

“I didn’t know that [exercise] was so important, I didn’t know at all, and I must sincerely say if I hadn’t listened to that bloke, I wouldn’t do it at all.” (Participant 6)

“That’s why I booked up for the exercising because of what he said … I did the exercising for the reason of this man, you know, convincing me I’ve got to do it.” (Participant 10)

This was not the case for others, though. There were some consistent characteristics of those who reported that the intervention had changed their behaviour. First, a relatively modest history of PA: they were not highly active but were not inactive either. Second, possession of a positive disposition:

“Every day’s a bonus, you know, and this operation has given me a bonus. As I said, life is sweet.” (Participant 10)

“I’m very positive about everything.” (Participant 6)

“Health doesn't seem to be a barrier to me. It’s all down to the right mental attitude and the right physical attitude and everything.” (Participant 1)

There was some indication that, for these participants, there might be a link between the positive message framing, and persuasion:

“[Interviewer: What was it about that talk that made you think, ‘I need to sign up for exercise’?] Surviving, and he [patient speaker] understands life is sweet without saying it.” (Participant 10)

##### Social comparison and attitude renewal

This theme describes a process articulated by patients who reported behavioural change as a result of the intervention. It can be described as a three-step process, in which (1) these patients compared themselves with the patient speaker, based on their empathic connection with him; resulting in (2) a revelatory moment; leading to (3) a renewed resolve to exercise.

Step 1. The personal story told by the patient speaker created a space in which the recipients experienced empathy stemming from their own personal experience.

“I could relate to him … You can put yourself in his shoes, because you feel exactly the same as he felt. Like I said, the physio was good, it was technical but with [patient speaker], I could be [him] at that moment because I could feel the same thing he felt.” (Participant 1)

“Here was a chap who had been in it and had come through. Not tearful as such, but quite poignant to hear his story.” (Participant 7)

Some participants described a social comparison process accompanying this empathic connection.

“You reflect on your own behaviour. It makes you think. You look at it, ‘Well I could do more. It's for my own good.’ You realise that, ‘Well I could do better than I'm doing.’” (Participant 1)

“I was saying to the guy next to me, I said, ‘He was the most important man here,’ … I said, ‘He’s an example.’ And, well, I listened to him and I do exactly what he said, you know, ‘Do your exercise.’ … It might sound a bit bad, but, oh, if he can do it, I can do it … that’s the way I was thinking” (Participant 6)

Step 2. These participants described this process as a revelatory moment.

“It was an eye opener to me, and I just went, ‘I'm doing the gym now.’” (Participant 2)

“It was a lightbulb moment.” (Participant 10)

Step 3. This process seemed to result in a renewed resolve to engage in PA.

“It affected me. The fact that I realised that if I want to be here I’ve got to be a little bit more proactive in my health and strength.” (Participant 2)

“It affected [my attitude toward PA] in a good way.” (Participant 6)

“Yes, it did [change my attitude toward PA]. It did work. Subconsciously, it worked.” (Participant 10)

## Discussion

Overall results from this pilot study indicate that the addition of a 10-min PA presentation, delivered by a previous PCa patient in the style of a personal story alongside a talk by a cancer exercise specialist, can result in benefits to quality of life, fatigue, and psychological wellbeing, in men who have undergone radical prostatectomy. Qualitative data imply that influence of the intervention on PA behavioural intention, in certain types of individuals, may involve social comparison processes and a revelatory moment, facilitated by an affective response to the intervention.

To the authors’ knowledge, this is the first study to investigate the physical and psychological effects of a peer support intervention for cancer patients in this manner, i.e. delivered by a former cancer patient; involving a single contact; and in a group setting. Other peer-support interventions for cancer patients have typically either used peer supporters that do not have cancer; or used intervention protocols that involve sustained contact with patients (for a recent scoping review, see Kowitt et al. [25]).

The most similar intervention the authors found was the subject of a 2016 mixed methods study by Ozier and Cashman, investigating a single one-to-one meeting between newly diagnosed brain tumour patients and a former brain tumour patient [26]. Qualitative themes from that study described their intervention as providing a sense of hope, alleviation of loneliness and appreciation of an authoritative source. These themes were broadly consistent with the themes reported here. The two studies suggest that benefits to psychological wellbeing provided by peer-support could benefit more patients via integration into the regular care pathway. This is particularly pertinent to the PCa population, given that men are less likely to engage with cancer support groups than women [27]. It may be useful to examine how such integrated practices could affect more rigorous measures of anxiety and depression, such as the Hospital Anxiety and Depression Scale (HADS) [28].

Some of the participants who were interviewed reported that the intervention had increased their PA, but these reports were not borne out in the quantitative data. There are some potential explanations for this. Self-reporting of PA is notoriously unreliable due to recall bias, social desirability effects, and individual variances in how the PA questionnaire is interpreted [29]. This may have reduced the sensitivity of the measure to genuine changes in behaviour. Another explanation may be that those reporting behaviour change in interviews were conflating behavioural intention with actual behaviour, which may not mean that they are increasing PA behaviour (i.e. the ‘intention–behaviour gap’ [30]).

Notable in this regard was the observed relationship between being exposed to the intervention, and subsequently reporting lesser problems with performing usual activities, and pain or discomfort. It could be observed that intervention participants also tended toward lesser mobility problems, but the overall number of men improving or deteriorating on this measure was small, which may have precluded statistical significance. These changes might be due to increased PA. Alternatively, intervention patients could be demonstrating response shift due to a modified psychological framing of their situation [31].

There are some limitations of this pilot study. First, the same person was used to deliver all the patient presentations. Second, some of the benefits described here may have limited transferability to other contexts (for example, cancer patients with non-curative disease). Third, none of the patients interviewed were physically inactive, which constitutes a sampling limitation regarding theoretical insights.

In conclusion, the addition to standard care of a single PA presentation for men with PCa, delivered by a former patient, may result in clinically meaningful benefits to quality of life at 100 days follow up. There may also be a role for such an intervention in influencing the PA intention of men with PCa.

## Electronic supplementary material

ESM 1 (DOCX 25 kb).

## Footnotes

1 Behaviour change techniques (BCTs) [20] applied under each condition are shown in Supplementary Table 1.
