# Supplementary material for: Effect of a brief physical activity-based presentation by a former patient for men treated with radical prostatectomy for prostate cancer: a mixed methods pilot study
Source: Support Care Cancer. 2020 Apr 22;29(1):145–54. doi: 10.1007/s00520-020-05455-4 (PMC7686188; doi:10.1007/s00520-020-05455-4)
Supplement: Supplementary file 1 — (DOCX 25 kb). [file 520_2020_5455_MOESM1_ESM.docx]

| **Control seminar** | | **Intervention seminar** | |
| --- | --- | --- | --- |
| Instruction on how to perform the behaviour |  | Instruction on how to perform the behaviour |  |
| Information about antecedents |  | Information about antecedents |  |
| Information about health consequences | Credible source (cancer exercise specialist) | Information about health consequences | Credible source (cancer exercise specialist) |
| Information about emotional consequences |  | Information about emotional consequences |  |
| Demonstration of the behaviour |  | Demonstration of the behaviour |  |
| Framing/reframing |  | Framing/reframing |  |
|  |  | **Information about health consequences** |  |
|  |  | **Information about emotional consequences** | **Credible source (patient)** |
|  |  | **Salience of consequences** |  |

Supplementary table 1

*Behaviour change techniques applied.*
